# Cytotoxicity of amide-linked local anesthetics on melanoma cells via inhibition of Ras and RhoA signaling independent of sodium channel blockade

**DOI:** 10.1186/s12871-020-00957-4

**Published:** 2020-02-21

**Authors:** Qinghong Zheng, Xiaohong Peng, Yaqin Zhang

**Affiliations:** Department of Anesthesia, Wuhan Fourth Hospital; Puai Hospital, Tongji Medical College, Huazhong University of Science and Technology, 473 Hanzheng Street, Qiaokou District, Wuhan, 430033 Hubei China

**Keywords:** Local anesthetics, Ras, RhoA, Voltage-gated sodium channel, Melanoma

## Abstract

**Background:**

Substantial clinical and preclinical evidence have indicated the association between amide-linked local anesthesia and the long-term outcomes of cancer patients. However, the potential effects of local anesthesia on cancer recurrence are inconclusive and the underlying mechanisms remain poorly understood.

**Methods:**

We systematically examined the effects of three commonly used local anesthetics in melanoma cells and analyzed the underlying mechanisms focusing on small GTPases.

**Results:**

Ropivacaine and lidocaine but not bupivacaine inhibited migration and proliferation, and induced apoptosis in melanoma cells. In addition, ropivacaine and lidocaine but not bupivacaine significantly augmented the in vitro efficacy of vemurafenib (a B-Raf inhibitor for melanoma with BRAF V600E mutation) and dacarbazine (a chemotherapeutic drug). Mechanistically, ropivacaine but not bupivacaine decreased the activities of Ras superfamily members with the dominant inhibitory effects on RhoA and Ras, independent of sodium channel blockade. Rescue studies using constitutively active Ras and Rho activator calpeptin demonstrated that ropivacaine inhibited migration mainly through RhoA whereas growth and survival were mainly inhibited through Ras in melanoma cells. We further detected a global reduction of downstream signaling of Ras and RhoA in ropivacaine-treated melanoma cells.

**Conclusion:**

Our study is the first to demonstrate the anti-melanoma activity of ropivacaine and lidocaine but not bupivacaine, via targeting small GTPases. Our findings provide preclinical evidence on how amide-linked local anesthetics could affect melanoma patients.

## Background

Melanoma is a highly aggressive skin malignancy with increasing incidence over the past decades [1]. The current treatment include radio-chemotherapy for early stage of melanoma, targeted therapy such as B-raf inhibitor vemurafenib for metastatic melanoma [2], surgery to remove the tumor at all stages of melanoma [3]. Several retrospective studies of patients undergoing cancer surgery indicate that the choice of anesthetic technique might translate into a clinical benefit such as prolonged survival after cancer surgery [4]. In particular, local anesthesia has been shown to reduce tumor metastasis and recurrence in patients undergoing surgery with breast or prostate cancer [5, 6]. Additionally, regional anesthesia in combination with or without general anesthesia would result in improved overall survival in patients with colorectal cancer [7].

In line with clinical observations, preclinical studies suggest that amide-linked local anesthetics have anti-tumor effects. Ropivacaine, lidocaine and bupivacaine are amide-linked local anesthetics and act on neuron cells via blocking voltage-gated sodium-channel (VGSC) and subsequent depolarization suppression [8]. They have been shown to exhibit anti-proliferative, anti-metastatic and pro-apoptotic potential on cell culture and xenograft mouse models in a variety of cancers [9–13]. In addition, local anesthetics preferentially target cancer stem cells [14]. Apart from their direct inhibitory effects on tumor cells, ropivacaine and lidocaine also negatively affect tumor microenvironment, such as angiogenesis [15, 16].

In this study, we thoroughly investigated the effect of ropivacaine, lidocaine and bupivacaine alone and their combination with anti-melanoma drugs on melanoma cell migration, proliferation and survival. We show that ropivacaine and lidocaine but not bupivacaine has anti-melanoma activity and acts synergistically with standard of care drugs in melanoma. We further demonstrate that the underlying mechanisms are via targeting RhoA and Ras signaling pathways, and this is in a VGSC blockade-independent manner.

## Methods

### Cell culture and drug reconstitution

Human melanoma cell lines A375 and A431 (Cell Lines Service, Germany) were cultured in RPMI 1640 medium (Invitrogen, US) supplemented with 2 mM glutamine and 10% heat-inactivated fetal bovine serum (Gibco, US). Ropivacaine and bupivacaine (Sigma, US) were dissolved in water and lidocaine was reconstituted in Hanks Balanced Salt Solution. Veratridine (R&D Systems, US), vemurafenib (LC Laboratories, US), calpeptin (Sigma, US) and dacarbazine (Selleckchem, US) were reconstituted in dimethyl sulfoxide (DMSO). Tetrodotoxin (Sigma, US) was dissolved in citrate buffer.

### Proliferation assay

5 × 10^3^ cells were seeded to each well in a 96-well plate. The next day, cells were treated with drugs at various concentrations for 72 h. Proliferation was measured using bromodeoxyuridine / 5-bromo-2′-deoxyuridine (BrdU) Cell Proliferation Assay Kit (Abcam, US) as per manufacturer’s protocols.

### Measurement of cell apoptosis and migration

Migration assay was performed using the Boyden chamber (Cell Biolabs Inc. US) with transwell inserts of 8 μm pore size as described in our previous study [17]. The migrated cells from five random fields were counted under the microscope (Zeiss, Germany). Apoptosis assay was assessed by flow cytometry of Annexin V staining as described in our previous study [13]. The treatment duration for migration and apoptosis were 8 h and 72 h, respectively.

### Western blot analyses

After 24 h drug treatment, total protein was extracted using lysis buffer contained 4% SDS, protease inhibitor cocktail and phosphatase inhibitor (Roche, US). Equal amount of total proteins was resolved using denaturing sodium dodecyl sulfate-polyacrylamide gel electrophoresis and analyzed by Western blot. Antibodies used in WB analyses include anti-p-MYPT1 (Cell Signaling, Cat. No.4563), anti-p-MLC (Cell Signaling, Cat.No.3671), anti-MLC (Cell Signaling, Cat.No.3672), anti-MYPT1 (Cell Signaling, Cat. No.2634), anti-p-Raf (Abcam, Cat. No. ab135559), anti-Raf (Abcam, Cat. No. ab137435), anti-p-ERK (Santa Cruz, Cat. No. sc-16,982), anti-ERK (Santa Cruz, Cat. No. sc-292,838), anti-Ras(Q61L) antibody (NewEast Biosciences, Cat. No. NEBA10195) and anti-β-actin (Santa Cruz, Cat. No. sc-130,656). Immunoblots shown in the accompanying figures are representative of three independent experiments.

### Measurement of RhoA, Rac1 and Ras activity

After 24 h drug treatment, cellular RhoA, Rac1 and Ras activities were assessed using total cell lysates and were measured using RhoA G-LISA Activation Assay Kit, Rac1 G-LISA Activation Assay Kit and Ras G-LISA Activation Assay Kit (Cytoskeleton Inc. US).

### Plasmid transfection

Cells were transfected with control plasmid (pSecTag2A vector) and pHras (Q61L). Constitutively active Ras (Q61L) was cloned to pSecTag2A from Addgene plasmid # 83186. Plasmid DNA transfection was performed using Lipofectamine 2000 transfection reagent (Invitrogen) as per the manufacture’s protocol. Cells were processed for cellular assays at 48 h post-transfection.

### Statistical analyses

All data are expressed as mean and standard error measurement (SEM) to indicate data variability. Comparisons of categorical variables by student t test or one way ANOVA were performed using Prism version 8.0 (GraphPad Inc., USA). *P*-value < 0.05 was defined as statistically significant.

## Results

### Ropivacaine and lidocaine but not bupivacaine demonstrates anti-migratory, anti-proliferative and pro-apoptotic effects to melanoma cells

**Fig. 1:**
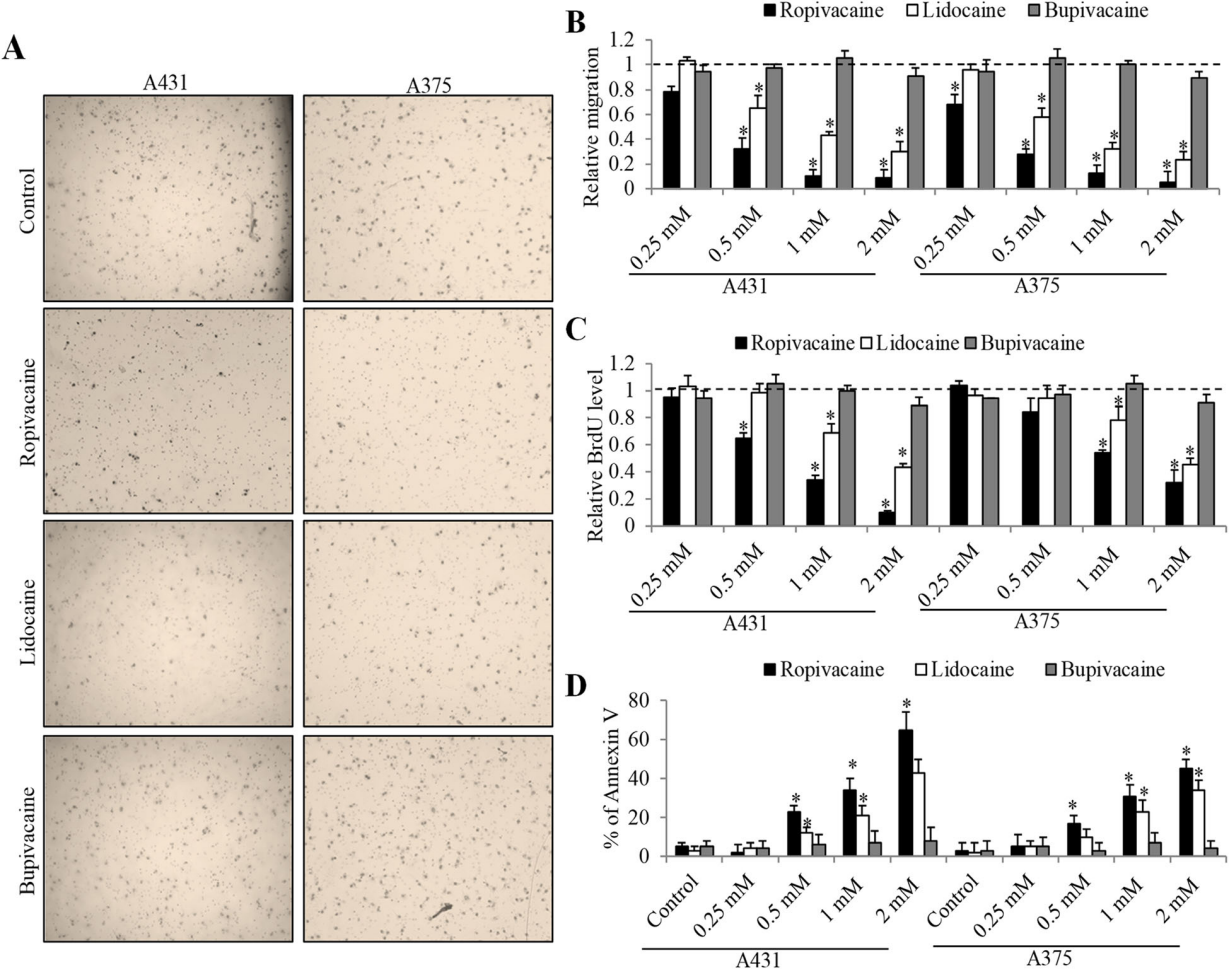
The inhibitory effects of local anesthetics on melanoma cell migration, growth and survival. (**a**) Representative images of melanoma cell migration in the absence and presence of 1 mM ropivacaine, lidocaine or bupivacaine. (**b**) Quantification of five random fields per sample using NIH ImageJ software shows the anti-migratory effects of ropivacaine and lidocaine but not bupivacaine in A375 and A431 cells. The differential effects of three local anesthetics at concentration range from 0.25 to 2 mM on melanoma cell proliferation (**c**) and survival (**d**). Annexin V-positive cells were considered as apoptotic cells. The data were derived from three independent experiments and presented as mean ± SEM. *p < 0.05, compared to control

We first analyzed the effects of three commonly used local anesthetics on melanoma cells migration, growth and survival. Two human cell lines modeling in vitro melanoma with varying cellular origin and genetic profiling were chosen in this study. A375 harbors BRAF V600E mutation and is p53 positive whereas A431 contains wildtype BRAF [18]. Ropivacaine, lidocaine and bupivacaine at concentration range from 0.2 to 2 mM were tested. As shown in Fig. 1a and b, and supplementary Figs. 1 and 2, ropivacaine and lidocaine significantly inhibited both A375 and A431 cell migration in a concentration-dependent manner. In addition, ropivacaine and lidocaine decreased proliferation as shown by BrdU level and induced apoptosis as shown by Annexin V percentage in melanoma cells (Fig. 1c and d, and supplementary Figs. 3 and 4). Notably, ropivacaine is more potent than lidocaine in melanoma cells. We also observed that the starting concentration (0.25 mM) required to inhibit migration is the lowest compared to the concentration (0.5 mM) needed to inhibit proliferation and induce apoptosis, suggesting that ropivacaine is more effective in inhibiting migration than growth and survival in melanoma cells. In contrast, bupivacaine up to 2 mM did not affect melanoma cell migration, growth or survival (Fig. 1).

### Ropivacaine and lidocaine but not bupivacaine augments the inhibitory effects of vemurafenib and dacarbazine in melanoma cells

**Fig. 2:**
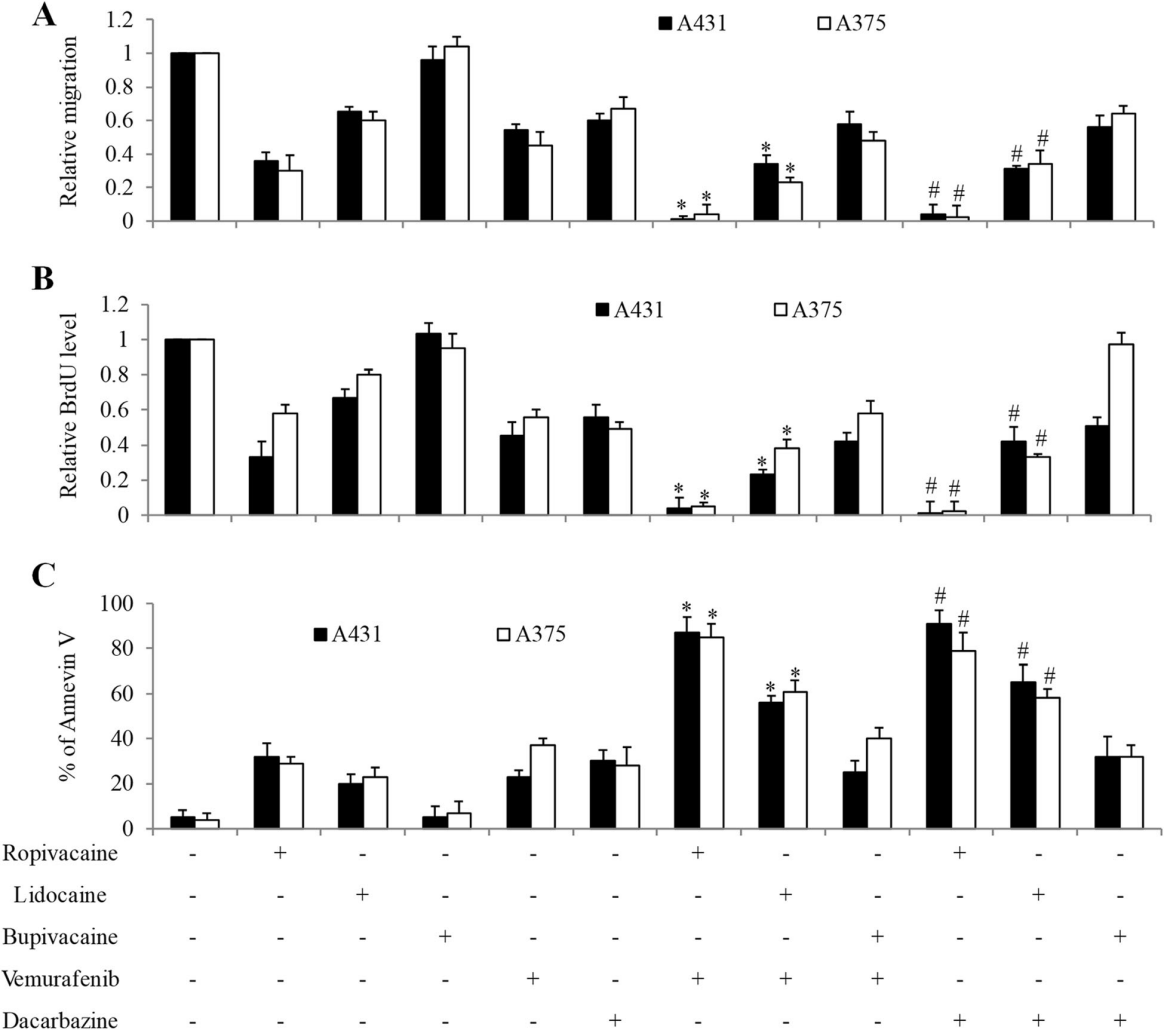
The combinatory effects of local anesthetics with vemurafenib and dacarbazine on melanoma cell migration, growth and survival. Ropivacaine and lidocaine but not bupivacaine significantly further enhanced the anti-migratory (**a**), anti-proliferative (**b**) and pro-apoptotic (**c**) effects of vemurafenib and dacarbazine. Vemurafenib at 1 μM and dacarbazine at 50 μM were used for the combination studies. Ropivacaine, lidocaine and bupivacaine at 0.5 mM, 1 mM and 1 mM were used for migration, proliferation and apoptosis assays, respectively. The data were derived from three independent experiments and presented as mean ± SEM. *p < 0.05, compared to vemurafenib; #*p* < 0.05, compared to dacarbazine

We next determined the combinatory effects of local anesthetics with drugs commonly used for melanoma treatment. Dacarbazine is a chemotherapeutic drug for metastatic melanoma [19] and vemurafenib is a B-Raf enzyme inhibitor to treat late stage of melanoma with BRAF V600E mutation [20]. The dose we had selected for combination studies is the dose that gives moderate effect as single drug alone. We found that ropivacaine and lidocaine significantly enhanced the in vitro efficacy of dacarbazine in suppressing migration and proliferation, and inducing apoptosis in melanoma cells (Fig. 2 and supplementary 5 to 8). Similarly, the combination of vemurafenib with ropivacaine or lidocaine is more effective than vemurafenib alone (Fig. 2). The combinatory effects of local anaesthetics with vemurafenib or dacarbazine are likely to be synergistic. For example, the Annexin V% in the combinatory group is more than the sum of Annexin V% in two single drugs. We did not observe further inhibition of the combination of bupivacaine with vemurafenib or dacarbazine in melanoma cells (Fig. 2). These results demonstrate that ropivacaine and lidocaine but not bupivacaine acts synergistically with both targeted therapy or chemo therapy drugs in melanoma cells.

### Ropivacaine but not bupivacaine inhibits GTPases activities in melanoma cells in a voltage-gated sodium channel (VGSC)-independent manner

**Fig. 3:**
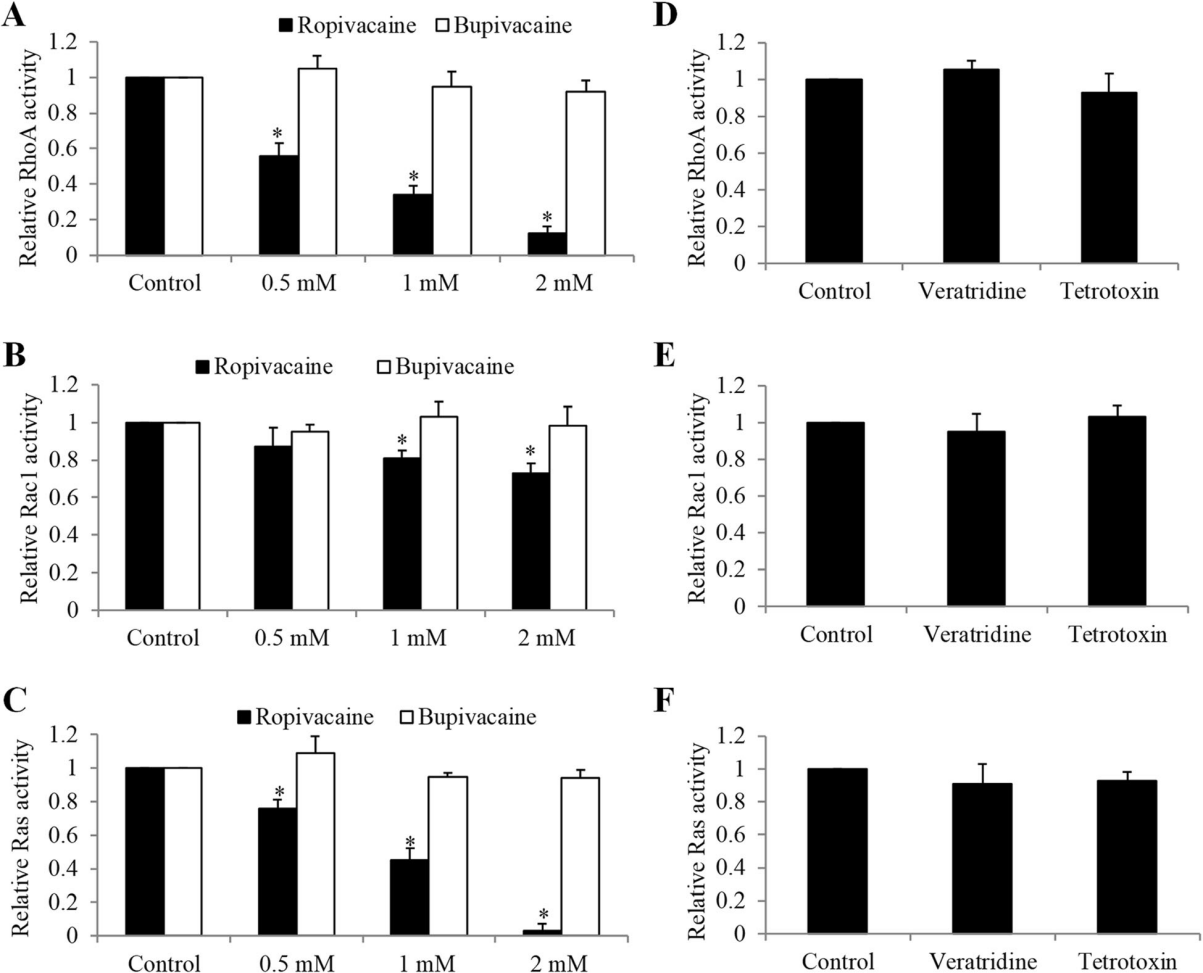
Ropivacaine but not bupivacaine or sodium channel inhibitor and activator decreased RhoA, Rac1 and Ras activities in melanoma cells. Ropivacaine but not bupivacaine significantly decreased RhoA (**a**), Rac1(**b**) and Ras (**c**) activities in A431 cells. Sodium channel activator veratridine (0.03 mM) and blocker tetrodotoxin (100 nM) did not affect RhoA (**d**), Rac1 (**e**) and Ras (**f**) activity in A431 cells. The data were derived from three independent experiments and presented as mean ± SEM. *p < 0.05, compared to control

RhoA, Rac1 and Ras are members of Ras super family of small GTPases that are critically involved in tumor cell biological activities such as migration, growth and survival [21]. Our previous study has revealed that ropivacaine inhibited esophageal carcinoma cells via targeting Rac1 [17]. To understand the molecular mechanism of ropivacaine’s action in melanoma cells, we investigated the effects of ropivacaine on small GTPases. We found that ropivacaine significantly decreased RhoA, Rac1 as well as Ras activities in melanoma cells (Fig. 3a to c). Similar to ropivacaine, we found that lidocaine also significantly decreased the activities of RhoA, Rac1 and Ras in melanoma cells (Supplementary Fig. S9). In contrast, bupivacaine which did not display inhibitory effects on melanoma cells did not affect RhoA, Rac1 and Ras activities (Fig. 3a to c), suggesting the specific inhibitory effects of ropivacaine on these small GTPases. Additionally, ropivacaine decreased RhoA and Ras activities to a larger extent than Rac1 activity, suggesting that the dominant effects of ropivacaine are inhibition of RhoA and Ras rather than Rac1 in melanoma cells.

We next determined whether the inhibitory effects of ropivacaine on small GTPases were associated with ropivacaine’s action on voltage-gated sodium channels (VGSC) [8]. We found that VGSC activator vetratridine at concentrations that abolished amide-linked local anesthesia-induced membrane depolarization [22] did not affect melanoma cell RhoA, Rac1 or Ras activity (Fig. 3d to f). Furthermore, VGSC blocker tetrodotoxin at the concentration that inhibits all VGSCs in excitable membranes [23] did not affect these small GTPases activities (Fig. 3d to f). The addition of tetrodotoxin did not abolish the inhibitory effects of ropivacaine on RhoA and Ras activities (Supplementary Fig. 10). These results suggest that the inhibitory effects of ropivacaine on small GTPases are not associated with sodium channel blockade.

### Ropivacaine acts on melanoma cells via inhibiting Ras and RhoA signalling pathways

**Fig. 4:**
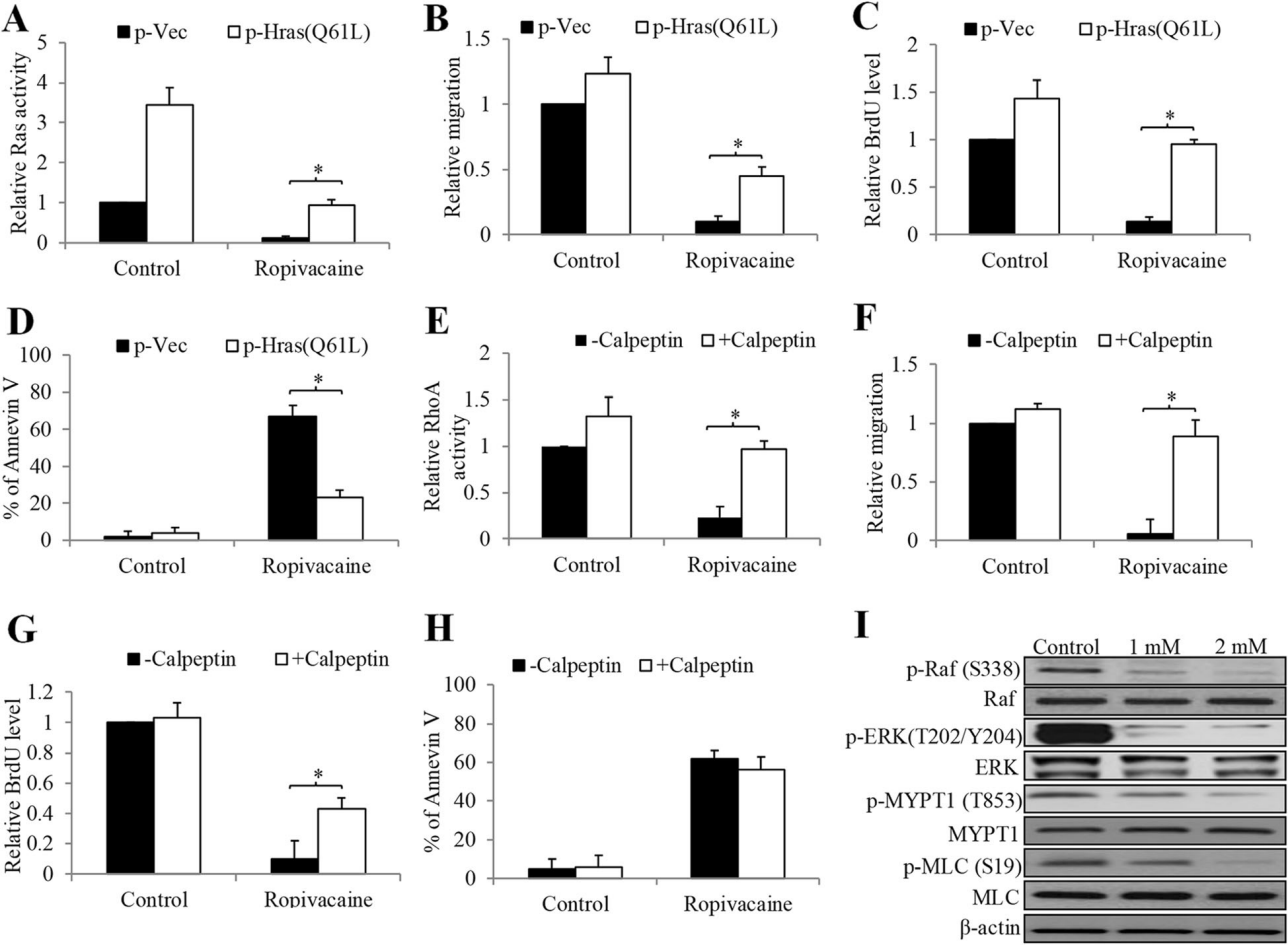
Ropivacaine’s inhibitory effects were abolished by active Ras overexpression or RhoA activator in melanoma cells. Overexpression of constitutively active Ras significantly reversed the effects of ropivacaine (2 mM) in decreasing Ras activity (**a**), inhibiting migration (**b**), decreasing BrdU level (**c**) and inducing apoptosis (**d**) in A431 cells. RhoA activator calpeptin significantly reversed the effects of ropivacaine (2 mM) in decreasing RhoA activity (**e**), inhibiting migration (**f**) and decreasing BrdU level (**g**) in A431 cells. (**h**) Calpeptin did not reverse ropivacaine’s effect in inducing apoptosis in A431 cells. (**i**) Western blot of A431 cells treated with ropivacaine for 24 h. Representative western blot photos were shown. *p < 0.05, compared to p-Vec or -Calpeptin

To confirm that ropivacaine acts on melanoma cells via targeting small GTPases, we attempted to rescue ropivacaine’s inhibitory effects using genetic and pharmacological approaches. We overexpressed constitutively active Ras (Q61L) in A431 melanoma cells and observed the increased mRNA and protein level of Ras (Q61L) as well as increased Ras activity (Fig. 4a and Supplementary Fig. 11). As expected, the decreased Ras activity by ropivacaine was rescued by Ras (Q61L) overexpression. Notably, we further found that overexpression of constitutively active Ras partially but significantly abolished the inhibitory effects of ropivacaine on melanoma cell migration, growth and survival (Fig. 4b to d), demonstrating that Ras inhibition is involved in ropivacaine’s ability in inhibiting melanoma cell migration, growth and survival. In addition, Rho activator I calpeptin [24] also partially but significantly reversed the anti-migratory and anti-proliferative but not pro-apoptotic effects of ropivacaine (Fig. 4e to h), indicating that RhoA inhibition is involved in ropivacaine’s ability in inhibiting melanoma cell migration and growth but not survival. Consistently, western blot analysis of phosphorylation level of the essential molecules downstream of Ras and RhoA signalling in cells exposed to ropivacaine demonstrated the decreased phosphorylation of Raf and MEK, MYPT1 and MLC (Fig. 4i), demonstrating that ropivacaine inhibits Ras/Raf/ERK and RhoA/MYPT1/MCL signalling pathways in melanoma cells.

## Discussion

In this present study, we found that ropivacaine and lidocaine but not bupivacaine resulted in significant inhibitions of cell migration and growth, and induction of cell apoptosis in melanoma. This is consistent with the previous study showing the cytotoxic effects of local anesthesia through lidocaine and ropivacaine on a human melanoma cell line [25]. Our study further extends the previous study by showing that 1) bupivacaine is not toxic to melanoma cells; 2) local anesthetics have differential effects on the varying biological activities of melanoma cells; and 3) the molecular mechanisms are via targeting small GTPases.

After treatment of ropivacaine and lidocaine but not bupivacaine at concentration range from 0.25 to 2 mM, we observed a significant reduction on the migrated cell number and BrdU level, and an increase in the percentage of Annexin V in two cell lines which represent human melanoma model with different cellular origin and oncogenic mutations (Fig. 1). The mean peak plasma concentrations of local anesthetics following transversus abdominis plane block is between 1 and 3 μM [26]. Similarly, Li et al’s work referred 0.02 to 0.1 mM as clinical relevance doses of local anesthetics [27]. The rational of testing concentration of local anesthetics that far exceeds the plasma concentration is because local anesthetics have wide range of uses in clinical practice and their plasma concentrations can vary widely. In addition, the surrounding tissues of tumor could be infiltrated with local anesthetic at the concentration range of clinical preparations. For example, the local infiltration concentration of ropivacaine can reach ~ 8 mM [27]. It is interesting to note that ropivacaine and lidocaine are more effective in inhibiting migration and growth than inducing apoptosis, suggesting that their anti-migratory and anti-proliferative effects are more pronounce in melanoma cells. This is supported by our previous study that ropivacaine potently inhibits esophageal cancer cell migration without affecting survival [17]. In addition, both ropivacaine and lidocaine significantly enhanced the in vitro efficacy of vemurafenib and dacarbazine in melanoma cells (Fig. 2). This is consistent with the previous work [13, 17], demonstrating the enhanced efficacy between amide-linked local anaesthetic and anti-cancer agents in cancer cells.

Although the anti-cancer activity of bupivacaine has been demonstrated in various cancers, including gastric cancer, prostate cancer and ovarian cancer [9, 28], our work and Li et al’s work demonstrate that bupivacaine does not affect melanoma migration, growth and survival [29]. In addition, bupivacaine does not inhibit breast cancer cell function [30]. Bupivacaine has been shown to augment chemotherapeutic agents’ efficacy in ovarian cancer and gastric cancer [9]. In gastric cancer model, we also demonstrated that bupivacaine acts synergistically with chemo drugs [17]. However, we did not observe any combinatory effects in melanoma cells when bupivacaine was combined with standard of care drugs for melanoma (Fig. 2). The differential effects observed in different types of cancers suggest that the anti-cancer activity of bupivacaine is cancer type-dependent.

The majority of melanoma cases demonstrate oncogenic activation of the KIT—NRAS—BRAF—MEK—ERK central axis that is a major regulator of cell differentiation and proliferation [31]. We identified that Ras and RhoA were the targets of ropivacaine in melanoma cells. Ropivacaine inhibited Ras and RhoA activities, and their global downstream signalling (Fig. 3 and 4a to c and i). The lack of changes in Ras and RhoA activities in melanoma cells following bupivacaine treatment (Fig. 3a to c) may also explain their unchanged migration, growth and survival behaviours. Particularly, we further revealed that ropivacaine inhibited migration mainly via suppressing RhoA whereas induced apoptosis mainly via inhibiting Ras in melanoma cells (Fig. 4a to h), and furthermore that this was not dependent on VGSC (Fig. 3d to f). As amide-linked local anesthetics, ropivacaine, lidocaine and bupivacaine act on neuron cells via blocking VGSC [8]. In our study, we found that ropivacaine acts on melanoma cells in a VGSC-independent manner. Other relevant studies including our previous work also demonstrate that the anti-cancer activities of local anesthetics are not through blocking VGSC [17, 32]. This might explain the differential activity of local anesthetics in cancer. We previously showed that ropivacaine targeted small GTPases via inhibiting prenylation in esophageal cancer cells [17]. Given our results that the activities of all tested small GTPases were affected by ropivacaine, we speculate that prenylation inhibition is likely to be involved in ropivacaine’s action in melanoma cells.

In conclusion, we have demonstrated a direct inhibitory effect of ropivacaine and lidocaine but not bupivacaine on melanoma cells, which are associated with sodium channel-independent inhibition of Ras and RhoA signaling (Supplementary Fig. 12). These findings indicate the differential effects of local anesthetics in cancer, depending on cancer types. Our findings provide experimental evidence and rationale to select the optimal anaesthetic regimens to further benefit melanoma patient care. However, we would also like to highlight that our work is not without limitations. Given the complexity in in vivo microenvironment and clinical settings, further large-scale prospective clinical trials are warranted to determine the effects of local anesthetics on longer-term reoccurrence or metastasis in patients with melanoma.

## Supplementary information

**Additional file 1 Figure S1.** The inhibitory effects of local anesthetics on melanoma cell migration. **Figure S2.** The inhibitory effects of local anesthetics on melanoma cell migration. **Figure S3.** The inhibitory effects of local anesthetics on melanoma cell survival. **Figure S4**. The inhibitory effects of local anesthetics on melanoma cell survival. **Figure S5.** The combinatory effects of local anesthetics with vemurafenib and dacarbazine on melanoma cell migration. **Figure S6**. The combinatory effects of local anesthetics with vemurafenib and dacarbazine on melanoma cell migration. **Figure S7.** The inhibitory effects of local anesthetics on melanoma cell survival. **Figure S8.** The inhibitory effects of local anesthetics on melanoma cell survival. **Figure S9.** Lidocaine decreased RhoA, Rac1 and Ras activities in melanoma cells. **Figure S10.** Tetrodotoxin does not abolish the inhibitory effect of ropivacaine in decreasing small GTPases activities in melanoma cells. **Figure S11.** Overexpression of Ras(Q61L) in A431 cells. **Figure S12.** The molecular mechanisms of ropivacaine’s action on melanoma.

## Data Availability

The datasets used and/or analysed during the current study available from the corresponding author on reasonable request.

## Abbreviations

BrdU: Bromodeoxyuridine / 5-bromo-2′-deoxyuridine
DMSO: Dimethyl sulfoxide
SEM: Standard error measurement
VGSC: Voltage-gated sodium-channel
