# Supplementary material for: Cytotoxicity of amide-linked local anesthetics on melanoma cells via inhibition of Ras and RhoA signaling independent of sodium channel blockade
Source: BMC Anesthesiol. 2020 Feb 21;20:43. doi: 10.1186/s12871-020-00957-4 (PMC7033929; doi:10.1186/s12871-020-00957-4)
Supplement: Supplementary file 1 — Additional file 1 Figure S1. The inhibitory effects of local anesthetics on melanoma cell migration. Figure S2. The inhibitory effects of local anesthetics on melanoma cell migration. Figure S3. The inhibitory effects of local anesthetics on melanoma cell survival. Figure S4. The inhibitory effects of local anesthetics on melanoma cell survival. Figure S5. The combinatory effects of local anesthetics with vemurafenib and dacarbazine on melanoma cell migration. Figure S6. The combinatory effects of local anesthetics with vemurafenib and dacarbazine on melanoma cell migration. Figure S7. The inhibitory effects of local anesthetics on melanoma cell survival. Figure S8. The inhibitory effects of local anesthetics on melanoma cell survival. Figure S9. Lidocaine decreased RhoA, Rac1 and Ras activities in melanoma cells. Figure S10. Tetrodotoxin does not abolish the inhibitory effect of ropivacaine in decreasing small GTPases activities in melanoma cells. Figure S11. Overexpression of Ras(Q61L) in A431 cells. Figure S12. The molecular mechanisms of ropivacaine’s action on melanoma. [file 12871_2020_957_MOESM1_ESM.doc]

**Cytotoxicity of amide-linked local anesthetics on melanoma cells via inhibition of Ras and RhoA signaling independent of sodium channel blockade**

**Supplementary Fig. 1: The inhibitory effects of local anesthetics on melanoma cell migration.** Representative images of A431 cell migration in the absence and presence of ropivacaine, lidocaine or bupivacaine.

**Supplementary Fig. 2: The inhibitory effects of local anesthetics on melanoma cell migration.** Representative images of A375 cell migration in the absence and presence of ropivacaine, lidocaine or bupivacaine.

**Supplementary Fig. 3: The inhibitory effects of local anesthetics on melanoma cell survival.** Representative flow cytometry dot plots showing the percentage of Annexin V and PI staining in A431 cells in the absence and presence of ropivacaine, lidocaine or bupivacaine.

**Supplementary Fig. 4: The inhibitory effects of local anesthetics on melanoma cell survival.** Representative flow cytometry dot plots showing the percentage of Annexin V and PI staining in A375 cells in the absence and presence of ropivacaine, lidocaine or bupivacaine.

**Supplementary Fig. 5: The combinatory effects of local anesthetics with vemurafenib and dacarbazine on melanoma cell migration.** Representative images of A431 cell migration in the absence and presence of ropivacaine (Rop), lidocaine (Lid) or bupivacaine (Bup) alone or in combination with vemurafenib (Vem) or dacarbazine (Dac).

**Supplementary Fig. 6: The combinatory effects of local anesthetics with vemurafenib and dacarbazine on melanoma cell migration.** Representative images of A375 cell migration in the absence and presence of ropivacaine (Rop), lidocaine (Lid) or bupivacaine (Bup) alone or in combination with vemurafenib (Vem) or dacarbazine (Dac).

**Supplementary Fig. 7: The inhibitory effects of local anesthetics on melanoma cell survival.** Representative flow cytometry dot plots showing the percentage of Annexin V and PI staining in A431 cells in the absence and presence of ropivacaine (Rop), lidocaine (Lid) or bupivacaine (Bup) alone or in combination with vemurafenib (Vem) or dacarbazine (Dac).

**Supplementary Fig. 8: The inhibitory effects of local anesthetics on melanoma cell survival.** Representative flow cytometry dot plots showing the percentage of Annexin V and PI staining in A375 cells in the absence and presence of ropivacaine (Rop), lidocaine (Lid) or bupivacaine (Bup) alone or in combination with vemurafenib (Vem) or dacarbazine (Dac).

**Supplementary Fig. 9: Lidocaine decreased RhoA, Rac1 and Ras activities in melanoma cells.** Lidocaine significantly decreased RhoA (A), Rac1(B) and Ras (C) activities in A431 cells. (D) Representative western blot image showing protein level of RhoA, Rac1 and Ras in A431 cells exposed to ropivacaine (2 mM) and lidocaine (2 mM). The data were derived from three independent experiments and presented as mean ± SEM. *p<0.05, compared to control.

**Supplementary Fig. 10: Tetrodotoxin does not abolish the inhibitory effect of ropivacaine in decreasing small GTPases activities in melanoma cells.** Tetrodotoxin (100 nM) did not reverse the decreased RhoA (A) and Ras (B) activities by ropivacaine (2 mM) in A431 cells. ns, not significant.

**Supplementary Fig. 11: Overexpression of Ras(Q61L) in A431 cells.** RT-PCR (A) and WB analysis (B) show overexpression of Hras (Q61) in cells transfected with p-Hras(Q61L) but not p-Vec. Both anti-his tag antibody and anti-Ras(Q61L) antibody were used in WB.

**Supplementary Fig. 12: The molecular mechanisms of ropivacaine’s action on melanoma.**
